# Supplementary figures and images for: Resolution dependency of sinking Lagrangian particles in ocean general circulation models
Source: PLoS One. 2020 Sep 10;15(9):e0238650. doi: 10.1371/journal.pone.0238650 (PMC7482921; doi:10.1371/journal.pone.0238650)

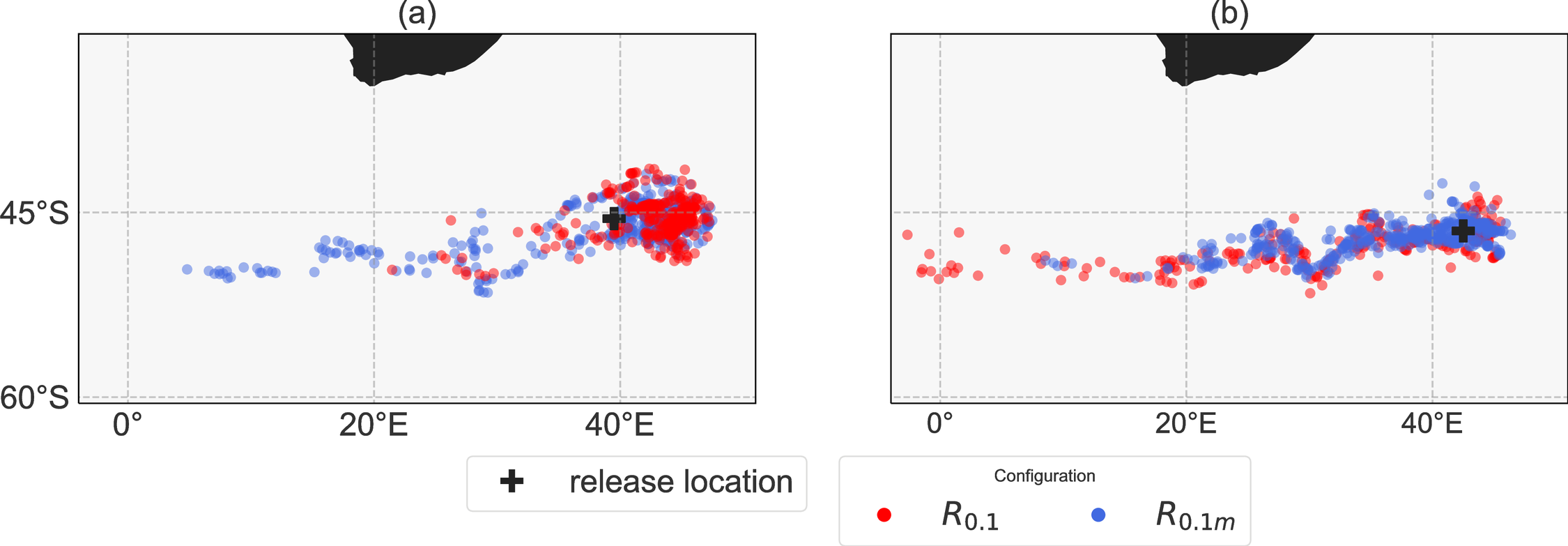

Supplement: S1 Fig — (a) 45.5°S, 39.5°E at 2068m depth (red on top of blue) (b) 46.5°S, 42.5°E at 2238m depth (blue on top of red). (TIF) [file pone.0238650.s001.tif]

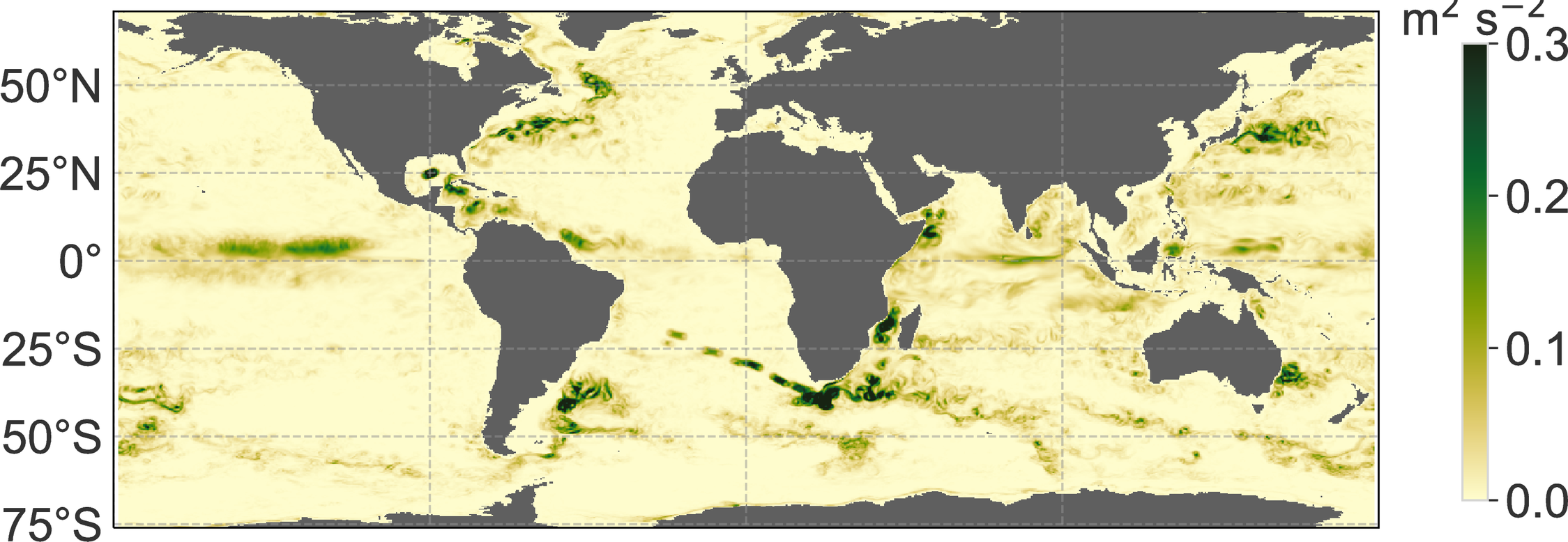

Supplement: S2 Fig — The eddy kinetic energy is defined as 12u′·u′¯, where the bar denotes the time mean and u′ the deviation from the time mean velocity vector u (so u(x→,t)=u¯(x→)+u′(x→,t)). (TIF) [file pone.0238650.s002.tif]
