# Supplementary material for: Resolution dependency of sinking Lagrangian particles in ocean general circulation models
Source: PLoS One. 2020 Sep 10;15(9):e0238650. doi: 10.1371/journal.pone.0238650 (PMC7482921; doi:10.1371/journal.pone.0238650)
Supplement: Attachment — Submitted filename: Review.pdf [file pone.0238650.s004.pdf]

Review of: *Resolution-dependent variations of sinking Lagrangian particles in general circulation models,* by P. D. Nooteboom et al.

This manuscript considers the problem of identifying the source location of sinking particles found at the bottom of the ocean and examines the effect of coarse spatial (and to some extent temporal) resolution. The authors find that ocean general circulation models (OGCMs) at a resolution typical of state-of-the-art paleoclimate applications (1 degree) do not capture the distributions of potential source locations well. This can be remedied to some degree by using a Smagorinski parameterisation for the eddy effects that are not directly modeled.

I find the experiment reasonably designed and the manuscript overall well written, although the organization is a bit odd. Some of the discussion could also benefit from a broader perspective, considering alternative explanations, and some of the conclusions are stated too strongly. Therefore, I recommend this submission for publication subject to **minor revisions**, which should address the points below.

Detailed comments:

1. Experiment design: The case with highest spatial and temporal resolution was chosen as the reference case. This is reasonable, since one might anticipate its results to be closest to being accurate. However, it makes it difficult to disentangle effects of spatial and temporal resolutions. You might want to consider using $R_{0.1m}$ as the reference case, which can then be compared to (a) a case with the same spatial and different temporal resolution and (b) cases with different spatial and the same temporal resolution.
2. Abstract, 4th sentence: State-of-the-art OGCMS run on regional grids nowadays often provide output at much greater resolution, e.g. 1 km in space and 3-hourly in time is fairly common. The authors may want to specify here (in the abstract) that they are considering global models only. It would also be good to discuss implications of the present findings for using a 10-km model with output every 5 days versus one with even higher spatial and temporal resolution. See also comment #22 below.
3. Abstract and Table 1: The phrase "5-daily" is not entirely clear, as it could mean 5 times per day or once every 5 days. I recommend rephrasing it to clarify in these places. Further mentions should then be self-explanatory.
4. Lines 22 – 27: There are some other published studies that seem to be relevant in this context, including e.g. Wekerle et al., *Frontiers Mar. Sci.*, 2018 (doi: 10.3389/fmars.2018.00407), which investigated sediment trap catchment areas using backward trajectories; Huntley et al., *Ocean Model.*, 2011 (doi: 10.1016/j.ocemod.2010.11.001), which looked at the impact of both temporal and

spatial resolution on the Lagrangian predictive skill of an OGCM for neutrally buoyant particles; Döös et al., *Ocean Model.*, 2011 (doi: 10.1016/j.ocemod.2011.05.005), which considered model-grid resolution impacts on dispersion rates; and Simonsen et al., *J. Enc. Radioactivity*, 2017 (doi: 10.1016/j.jenvrad.2017.06.002), which studied the effect of model resolution on the estimates of radionuclide transport.

5. Line 59: Particles are released every three days over what time period? (The caption of Fig. 1 suggests 6 years.)
6. Lines 106 – 109: This description is a bit unclear. First, there seem to be 9 different model configurations, depending on whether the choice for c_s is considered part of the configuration. I suggest to use 4 main configurations, whereby R_{1m} should consistently be chosen with the same value of c_s (Given the results, I would suggest $c_s = 2$), with 5 additional experiments to investigate the sensitivity of the results to c_s. It is not helpful that in the figures R_{1m} does not consistently refer to the same model configuration. Secondly, line 108 "backtracking from a single release location" appears to contradict line 61, which specifies a grid of release locations. Reading further, it becomes clearer what is meant, but I recommend editing the description here. Also, is the 130 particles used at each location a typo? For releases every 3 days for 6 years (cf. caption of Fig. 1), there should be 730 releases.
7. Lines 110 – 117, Fig. 1, and Results organization: On these lines and in the figure, the three metrics are listed in an order of increasing complexity. The lateral distance is a metric of an individual trajectory; the area metric describes the variability among launches at a single location; and the Wasserstein distance also takes into consideration where in space the distribution sits. This makes sense. But the Results are organized in the opposite order, starting with the most complex metric. I highly recommend changing that and following the order presented on lines 110 – 117, making it easier for the reader to interpret the additional elements captured in each metric.
8. Line 127: Could you provide the range of typical travel times for each sinking velocity? In other words, what travel time is too short or long enough to see the eddy effects?
9. Line 130: Well, they are not the same; one can clearly distinguish them in Fig. 1d. It might be better to state that they are not significantly different. Also, if you consider these two curves to be "the same", then so are the curves for the larger sinking velocity, which is not how they are discussed in the next paragraph.
10. Lines 131 – 134: I recommend explaining this earlier, maybe at the beginning of the Wasserstein distance comparison section, before the reader looks at the plot and starts wondering why that quantity isn't identically 0.
11. Line 136: There seems to be a strong difference between $c_s = 0$ and c_s positive. It is not clear that $c_s = 2$ is significantly different from the other positive c_s values. With that in mind, the statement that there is a minimizing value is misleading.
12. Line 147: Consider showing a plot of the geographic distribution of the eddy kinetic energy in the high resolution model to support this statement.

13. Lines 152 – 153: This sentence is unclear. "More likely" than what? Than in the other model? Than when the particles aren't near each other? Since the latter is obvious, I assume you meant the former. A clearer wording might be "Nearby particles are more likely to follow similar pathways in $R_{0.1m}$ than in $R_{0.1}$."
    However, it is also not clear how this observation is relevant to the experiment at hand. The particles within each analysed cluster are not "located close to each other", since they are separated in time. The link needs to be made more explicitly.
14. Line 155: At this point, the reader (or at least I) starts to wonder why $R_{0.1m}$ isn't included in Fig. 3. I suggest referencing Fig. 5 at this point.
15. Fig. 3: Why is c_s = 5 used here for panel (c)? It would help to pick a consistent value for all figures showing R_{1md}. Also, Fig. 3d suggests that a value of 3.5 would be best to approximate $R_{0.1}$, or a value of 2 to approximate $R_{0.1m}$. This is consistent for both sinking velocities. Could you comment on this?
16. Lines 177 – 179: Maybe I misunderstand what is meant by a "more smoothed pattern", but I don't think that smoothness explains this. Rather it is the lowering of the peaks in the Southern Ocean.
17. Lines 189 – 190: See comment #13 – it is not clear that this is relevant. The explanation might be instead that particles don't travel as far and hence don't spread out as much. That would be supported by Fig. 5b. This, in turn, is a result of the velocities generally having lower peaks due to longer temporal averaging.
18. Lines 193 – 194: The two panels of Fig. S1 should opposite behavior in a very similar location. In one case, the blue particles are closer together than the red ones, in the other they are farther apart. This needs to be addressed in the text.
19. Lines 197 – 200: This is not entirely true. Fig. 4 shows that in some regions (mainly the Southern Ocean), individual trajectories disperse *more* in the absence of eddies.
20. Fig. 6: It seems that R_{1m} appears mostly in black rather than yellow, because only the outlines of the markers are visible.
21. Lines 242 – 247: These conclusions are overstated. I am not convinced that the mean flow field in coarser models is wrong in *most* areas (line 242). At a minimum, this claim needs a reference.
    In addition, in Fig. 3 and Fig. 4, it looks like the diffusion *is* sufficient in *most* areas! So, I don't understand the justification for the claim in lines 246 – 247.
    Lastly, these impacts seem to be highly dependent on the sinking velocity, which needs to be acknowledged.
22. Lines 248 – 253: Will eddying (i.e., a resolution of ~ 10 km) suffice? Why wouldn't we expect this effect to continue as the model resolution is increased and eddies of smaller and smaller size are being resolved?
23. Lines 256 – 257: What does it mean that these models do not produce a flow field that is representative? I would imagine that paleo modelers would consider their models to represent the time period being modeled.

Typos and such:

- Abstract, 4th sentence: It should be "on the order of…"
- Line 35: There should be no comma before the "and".
- Line 61: "on a 1° x 1° global grid"
- Line 63: Since this isn't being shown in the present study, past tense would be better: "has been shown to be a proper way."
- Line 68: Again, it should be "on the order of…"
- Fig. 1 caption, last line: The "of" following the parentheses is superfluous.
- Fig. 2 caption, last sentence: "… is shown between the particle distributions of the same…"
- Fig. 2(c) title: "diffusion" is misspelled.
- Line 150: The reference should be to Fig. 1c (not b).
- Captions for Fig. 3 and Fig. 4: The subscript in c_s isn't written as a subscript. R_{1m} is missing the 'm'.
  For Fig. 4 caption, it should be "Globally averaged".
- Line 162: It should be "monotonically", not "monotonously".
- Lines 175 and 177: Replace "more smoothed" with "smoother".
- Line 187: Replace "more clear" with "clearer".
- All the global geographic plots in Fig. 2 – 4 have incorrect longitude labels. Also, consider using the same view for all global geographic plots, either that used in Fig. 5 or that used for the other figures.
- Fig. 6 caption, first line: R_{1m} is missing the 'm'.
  Second to last line: Are the depths really identical for panel (c), or is this a typo?
  Period missing at the end of the caption.
- References:
  a. Please include DOIs wherever possible.
  b. Ref. #4 should have second author "Nöthing E-M".
  c. Ref. #5 is missing the author's initial.
  d. Ref. #8 should have last author "van Sebille, E"; similar for Ref. #16.
  e. Ref. #13 should capitalize "García".
  f. Ref. #14 accidentally repeats part of the title.
  g. Ref. #44: If this is a book title, it shouldn't be abbreviated.
  h. Ref. #50 should capitalize "Kemper".
