# Supplementary material for: Resolution dependency of sinking Lagrangian particles in ocean general circulation models
Source: PLoS One. 2020 Sep 10;15(9):e0238650. doi: 10.1371/journal.pone.0238650 (PMC7482921; doi:10.1371/journal.pone.0238650)
Supplement: Attachment — Submitted filename: Reponse_to_reviewers.pdf [file pone.0238650.s005.pdf]

Editor

Thank you for submitting your manuscript to PLOS ONE. After careful consideration, we feel that it has merit but does not fully meet PLOS ONEs publication criteria as it currently stands. Therefore, we invite you to submit a revised version of the manuscript that addresses the points raised during the review process. It is particularly important for the authors to identify more clearly the audience they target with this work, and that they carefully revise the manuscript accordingly. Please read carefully the suggestions made by the reviewers so that they are addressed in the revised version.

We would like to thank the editor for consideration of this manuscript.

We would also like to point out that the manuscript is intended for a broad audience. The results in the manuscript have implications for e.g. modellers, sedimentologists, environmental marine scientists, biologists, paleontologists and oceanographers that use sinking Lagrangian particles in Eulerian models to track e.g. biogenic matter that is important for the carbon flux to the ocean bottom [12, 13, 29], microplankton which is used for climate reconstructions [14, 26] and sinking plastic [11]. We have adapted the manuscript accordingly and hope that the intended audience becomes clear.

We asked for a clarification to plosone@plos.org about the copyright of the figures with maps. We understand that the Cartopy code falls under GNU license, but Cartopy generated figures do not. As a consequence, Cartopy generated figures can be published under CC by 4.0. In most figures, we only used our own data to create the plot. However, figures 6 and S1 do use data from Natural Earth. Natural Earth is one of the suggestions of PLOS to create figures, and can be published under CC by 4.0.

Please find our replies to the reviewers and the points that are changed in the revised manuscript below.

On behalf of the authors,

Peter Nooteboom

Reviewer 1

We would like to thank the reviewer for his/her careful reading and his/her constructive comments.

Please find our point by point reply and the changes in the manuscript below.

On behalf of the authors,

Peter Nooteboom

This paper is a numerical discussion of particle motion in ocean general circulation models, principally as a function of spatial resolution. The conclusions are unsurprising: eddy resolution makes a big difference to long-term particle displacements. My inference is that the paper is directed *not* at numerical modelers, but at paleoclimate people who over-interpret results of crude circulation models. If this inference is correct, it should be made explicit in the abstract, introduction, and probably the title and raises the question of whether it belongs in a paleo-journal. If the intended audience is not the technically adept numerical modelling community, it is essential to tell the reader that the paper represents errors in Lagrangian displacements in *Eulerian* models. Non-expert readers should be told that although not common, Lagrangian circulation models exist, as do whole books on the subject (e.g., Bennett's). An interesting, technical, question would be how well a Lagrangian GCM can calculate the Eulerian mean flows.)

Author's response

Since the intended audience is indeed broader than the numerical modelling community, we added in the introduction that Lagrangian displacements in Eulerian models are represented. Whether Lagrangian GCMs calculate Eulerian mean flows well is indeed an interesting question which is out of scope of this paper. In this manuscript, however, we do investigate the resolution dependency of Lagrangian particles in Eulerian models.

Changes in manuscript

In the introduction, we changed a sentence:
'In this paper, we will assess how the sinking Lagrangian particle trajectories vary for different temporal or spatial resolutions of an Eulerian OGCM.'

We changed a sentence in the abstract to mention the audience explicitly, and to whom the results in this paper apply:
'We recommend to ~~apply~~ use sinking Lagrangian particles, representing e.g. marine snow, microplankton or sinking plastic, only with velocity fields from eddying Eulerian OGCMs, requiring high-resolution models in e.g. paleoceanographic studies.'

We stated in the abstract that the effect of eddies in OGCMs on the time mean flow is not new in physical oceanography:
State-of-the-art OGCMs are strongly eddying, similar to the real ocean, providing results with

a spatial resolution in on the order of 10 km on a daily frequency. Many While the importance of eddies in OGCMs is well-appreciated in the physical oceanographic community, other marine research communities may not. Further, many long term climate modelling simulations (e.g. in paleoclimate) rely on lower spatial resolution models that do not capture mesoscale features. It remains unclear To demonstrate how much the absence of mesoscale features in low-resolution models influences the Lagrangian particle transport. In this study, we simulate the transport of sinking Lagrangian particles using low- and high-resolution global OGCMs, and assess the lateral transport differences resulting from the difference in spatial and temporal model resolution.

We revised a paragraph in the introduction, to make explicitly clear which audience we target. We changed the following paragraph:
'In this paper, we will assess how the sinking Lagrangian particle trajectories vary for different temporal or spatial resolutions of an the Eulerian OGCM. We investigate the effect of eddies on the particle trajectories. Moreover, we study whether a stochastic lateral diffusion of Smagorinsky [22] type could parameterise the effects of the eddies in the non-eddying OGCM. We apply this analysis to an example of sinking microplankton used in [14] (specifically focusing on use the same analysis as is applied in [14] about microplankton (specifically dinoflagellate cysts) which is used for palaeoceanographic reconstruction. The results concern any type of application with sinking Lagrangian particles, such as the comparison of sediment trap data with OGCMs [29, 12] or the representation of sinking microplankton [26, 14] and sinking plastic [11].'

The title has been changed to *'Resolution dependency of sinking Lagrangian particles in ocean general circulation models'* to make the manuscript more appealing to a broader audience.

We added a sentence in the discussion:
'In turn, it should be appreciated that a regional paleoceanographic signal could be influenced by flow characteristics which are not represented by the non-eddying models. This represents a cautionary tale in putting too much confidence in flow fields from low-resolution fully coupled GCM simulations.'

The explanation of the website has been changed in the discussion, to stress more that the results are to be expected for a physical oceanographer, but important to appreciate by other communities:
'This study reveals the importance of eddying model simulations for sinking Lagrangian particles. The simulations of sinking particles in eddying OGCMs allow for the assessment of the surface origin location of sedimentary particles before they sink to the bottom. Hereby we launch planktondrift.science.uu.nl to disseminate these The effect of eddies on the flow should be appreciated outside the physical oceanography community. In order to facilitate increased understanding on this matter, interactively disseminate our results, and allow users to self-explore and verify the surface-ocean location of origin for sedimentary particles., we developed the website planktondrift.science.uu.nl containing our results.'

Why is a backwards in time calculation done? Does some stability issue not arise?

Author's response

We used backward in time calculation to be consistent with the studies in [26, 14]. These studies focus on particles which are found at the bottom of the geological ocean.

Changes in manuscript

To make clearer that we perform the backward calculation and stay consistent with [14, 26], we changed this sentence in the method section:
'We apply the same particle tracking approach as in [14]. This means that we release particles at the bottom of the ocean every three days for more than a year, and compute their trajectories in the changing flow field back in time (similar to [14, 25, 29, 12]) until the particles reached the surface.'

Again, for non-specialist audiences, it would be helpful to point out that skill in calculating the distribution of scalar tracers, such as temperature might be much more accurate in the parameterized Eulerian models—given how the parameterizations are constructed.

Author's response

Good point.

Changes in manuscript

We changed the sentence in the discussion:
'The bolus velocity is weaker compared to the Eulerian flow velocity (typically v_b is approximately 5% of v_a at the surface layer)~~, and it~~. Parameterisations like GM improve the temperature and salinity distribution in Eulerian models. GM is a type of 'extra advection,' which assumes that tracers mix along surfaces of contstant potential density [6]. However, GM does not make a relevant difference ~~at the time scales on which we apply the Lagrangian particles here~~if Lagrangian particles are applied offline to track other passive tracers. '

Reviewer 2

We would like to thank the reviewer for his/her careful reading and his/her constructive comments.

Please find our point by point reply and the changes in the manuscript below.

On behalf of the authors,

Peter Nooteboom

The manuscript describes how statistics of Lagrangian sinking particles differ when advected off-line in an eddy permitting or coarse resolution ocean model. While realizing the need for the work presented for some communities (paleo?), from a physical oceanography point of view, the content is not novel and mostly qualitative. It is well known that ocean models that are not eddy resolving underestimate horizontal dispersion. The literature on the subject is very large and some references should indeed be added. I note also that the manuscript in its current form reads like a physical oceanography work, and the appeal to the broader and more biologically oriented community of PLOS One is unclear to me.
I do not have major comments on what is in the manuscript (there is nothing wrong per se), but I would have hoped for a more in-depth justification of the work and its limitations considering the lack of novelty, or a more in-depth investigation of the reliability of the high and low resolution results. The outcome of this work confirms deductions that have been made already in the absence of sinking, and many Lagrangian studies have been performed in this regard comparing runs at the exact resolutions used here (to mention few relevant to the content of this manuscript: Putman and He, 2013; Doos, Rupolo and Brodeau, 2011, and many intercomparison papers focused on the Eulerian fields).

Author's response

We would like to point out that the manuscript is intended for a broad audience, since the results are relevant for work that concerns the application of sinking particles in Eulerian models to biogenic matter that is important for the carbon flux to the ocean bottom [12, 13, 29], microplankton which is used for climate reconstructions [14, 26] and sinking plastic [11]. We have adapted the manuscript accordingly and hope that the intended audience becomes clear.

Changes in manuscript

We changed a sentence in the abstract to mention the audience explicitly, and to whom the results in this paper apply:
'We recommend to ~~apply~~ use sinking Lagrangian particles, representing e.g. marine snow, microplankton or sinking plastic, only with velocity fields from eddying Eulerian OGCMs, requiring high-resolution models in e.g. paleoceanographic studies.'

We stated in the abstract that the effect of eddies in OGCMs on the time mean flow is not

new in physical oceanography:
State-of-the-art OGCMs are strongly eddying, similar to the real ocean, providing results with a spatial resolution ~~in~~ on the order of 10 km on a daily frequency. ~~Many~~ While the importance of eddies in OGCMs is well-appreciated in the physical oceanographic community, other marine research communities may not. Further, many long term climate modelling simulations (e.g. in paleoclimate) rely on lower spatial resolution models that do not capture mesoscale features. ~~It remains unclear~~ To demonstrate how much the absence of mesoscale features in low-resolution models influences the Lagrangian particle transport~~. In this study~~, we simulate the transport of sinking Lagrangian particles using low- and high-resolution global OGCMs, and assess the lateral transport differences resulting from the difference in spatial and temporal model resolution.

We revised a paragraph in the introduction, to make explicitly clear which audience we target. We changed the following paragraph:
'In this paper, we will assess how the sinking Lagrangian particle trajectories vary for different temporal or spatial resolutions of ~~an~~ the Eulerian OGCM. We investigate the effect of eddies on the particle trajectories. Moreover, we study whether a stochastic lateral diffusion of Smagorinsky [22] type could parameterise the effects of the eddies in the non-eddying OGCM. We ~~apply this analysis to an example of sinking microplankton used in [14] (specifically focusing on~~ use the same analysis as is applied in [14] about microplankton (specifically dinoflagellate cysts) which is used for palaeoceanographic reconstruction. The results concern any type of application with sinking Lagrangian particles, such as the comparison of sediment trap data with OGCMs [29, 12] or the representation of sinking microplankton [26, 14] and sinking plastic [11].'

The title has been changed to *'Resolution dependency of sinking Lagrangian particles in ocean general circulation models'* to make the manuscript more appealing to a broader audience.

The reference of Doos et al. 2011 [5] is used in the introduction:
'The spatial resolution of the OGCM determines if the flow is eddying, which played an important role in simulations of sinking particles near the northern Gulf of Mexico [12] and in the Benguela region [13], and for passive tracers near Sellafield [21] and globally [5] (0.25° versus 1° resolution).'

The reference of Putman and He 2013 [15] is used in the discussion:
'The model output of configuration $R_{0.1}$ could be coarsened to a 1° grid before the backtracking analysis, to separate out the effects on the Lagrangian analysis of (a) a coarsened grid (see also [10, 15]) and (b) a lower resolution of the underlying Eulerian model.'

We added a sentence in the discussion:
'In turn, it should be appreciated that a regional paleoceanographic signal could be influenced by flow characteristics which are not represented by the non-eddying models. This represents a cautionary tale in putting too much confidence in flow fields from low-resolution fully coupled GCM simulations.'

The explanation of the website has been changed in the discussion, to stress more that the

results are to be expected for a physical oceanographer, but important to appreciate by other communities:
‘~~This study reveals the importance of eddying model simulations for sinking Lagrangian particles. The simulations of sinking particles in eddying OGCMs allow for the assessment of the surface origin location of sedimentary particles before they sink to the bottom. Hereby we launch planktondrift.science.uu.nl to disseminate these~~ The effect of eddies on the flow should be appreciated outside the physical oceanography community. In order to facilitate increased understanding on this matter, interactively disseminate our results, and allow users to self-explore and verify the surface-ocean location of origin for sedimentary particles~~.~~, we developed the website planktondrift.science.uu.nl containing our results.’

In detail: Firstly, the manuscript never mentions the resolution limitations of the simulation used. 10km is not enough to resolve eddies at high latitudes, therefore the run is eddy permitting. No mention is made of the scales that are not resolved (effectively 30 km and below) in the high resolution case. Nothing is said about the vertical resolution (work by Stewart et al, 2017 and Bracco et al., 2018, both in Ocean Modelling may be of interest). In other words, can we expect to see similar results if we had a 1km resolution ocean model or 100 vertical layers? Accounting for work by, for example, Patrice Klein and others, the likely answer is no. Global runs at 2km have been made and are potentially freely available for analysis (MIT model, OFES).

Author’s response

Indeed, a comparison of the back-tracking analysis with Eulerian models of higher horizontal resolution and different vertical resolution is interesting. Especially, it can be interesting for the modelling of sinking particles in the present-day ocean. It is not entirely clear how much of a difference it would make globally.
The two model resolutions (1° and 0.1° horizontally) in this manuscript are respectively the highest resolutions that are typically used in palaeo and present-day models respectively. Due to long spin-up times, palaeo simulations at 10km resolution do not exist yet, and palaeo simulations at 2km resolution would be unfeasible.
However, this issue is now addressed in the discussion, as it is interesting for future work.

Changes in manuscript

We added a sentence in the method section to be clear about the choice of the model resolutions:
‘These configurations represent the differences between state-of-the-art, global OGCM resolutions of the past (1° horizontally and monthly model output) and the present-day (0.1° horizontally and model output on a daily scale). The single effect of model output with lower temporal resolution compared to the state-of-the-art present-day OGCMs is investigated in a separate configuration $R_{0.1m}$.’

Two paragraphs in the discussion are added now, which also include the references to Stewart el al. 2017 [23] and Bracco et al. 2018 [4]:
‘Future work could investigate the sinking Lagrangian particles in other configurations with different OGCM resolutions. The model output of configuration $R_{0.1}$ could be coarsened to a

1° *grid before the back-tracking analysis, to separate out the effects on the Lagrangian analysis of (a) a coarsened grid (see also [10, 15]) and (b) a lower resolution of the underlying Eulerian model. Moreover, particle trajectories could be sensitive to the vertical resolution of the OGCM (e.g. [4, 23]).*
Some applications require different horizontal model resolutions than the configurations used in this paper. An OGCM with a ∼*1km horizontal resolution has a better representation of the spatial/temporal submesoscale that can be important for sinking Lagrangian particles which represent the carbon flux to the ocean bottom [3]. Although the mesoscale flow contains most of the energy that is responsible for the horizontal tracer dispersion [24], submesoscale (1-20km) dynamics have proven to be of importance for the vertical advection of iron in specific regions with strong flow-bathymetric interactions [18].*'

Secondly, what would happen is a reanalysis data-set was used instead? The most recent ones are run at 25km-¿10km near the poles and fields are saved monthly or more frequently. Do the statistics in the 10km run compare well with those from a reanalysis product, where at least surface eddies are constrained by satellite observations and the water column dynamics assimilate the large ARGO dataset? The investigation of the time-dependence of the results (advecting particles using fields saved every month or every 5 days is also superficial and should be expanded).

Author's response

The suggested comparison with sinking particle statistics in reanalysis data is interesting, but outside the scope of this paper. Here, we focus only on the differences in spatial resolution and not the type of Eulerian model.
We chose to study the dependence of 5-daily and monthly output fields, because this is the typical timescale difference of model output between present-day and palaeomodels. For an analysis of 3D Lagrangian particles with more temporal resolutions we cite [16].

Thirdly, I understand the argument for justifying the simulation done for small plastic particles, but as far as I know organic particles small enough to have a sinking velocity of 6-20 m/day near the surface do not make it to the ocean bottom as such (vice versa particles that may reach the bottom with these sinking velocities were likely much larger and heavier at formation). Aggregation, grazing and bacterial activities are dominant processes on those sizes. The review by Boyd et al. in Nature in 2019 has a nice image summarizing the fate of sinking particles as function of their size, and shows that sinking is not the dominant process determining the fate of particles as small as those used in the manuscript (they do not reach more than 400-600 m depth in particle form). It is unclear, therefore, what is the paleo relevance that justifies the need for coarse resolution ocean models.

Author's response

The review in [3] is mainly about the processes which are responsible for the carbon flux to the ocean bottom. As measured in e.g. [17], the fast sinking particles contribute most to the carbon flux, however slow sinking particles contribute significantly to the number flux. As explained in [3], the mechanisms that are responsible for this carbon flux vary in space and time. As a

consequence, the slowly sinking particles can contribute significantly to the carbon flux [27, 1].

It could be interesting to make the sinking speed dependent on dynamics such as aggregation, grazing and bacterial activities. As such, one could obtain a more detailed and realistic sinking of particles. However, we chose to keep the sinking constant to get a better idea of the resolution dependency of these results, instead of the sensitivity of the results to parameters which determine the sinking speed.

Finally, the sinking particles that are used for climate reconstructions are not necessarily subject to the mechanisms explained in [3], which increase the sinking speed of small particles, as is explained for dinoflagellate cysts in [14]. Therefore, particles which are sinking at velocities on the order of 6-25 m/day are relevant for these palaeoclimate reconstructions. For an analysis of the effect of sinking velocities on the particle trajectories, we refer to [14].

Changes in manuscript

Reference [3] is cited in the discussion, to explain the relevance of future work on sinking particles in OGCMs of 1km scale resolution (see 'changes in manuscript' under the first detailed comment; page 6).

Overall, in my opinion this is an exercise that deserves publication if better motivated, with a more in-depth discussion of implications and limitations, and the exploration of a greater range of sinking velocities, saving times, etc.

Author's response

Modification in the paper based on the reviewer's comments led to a more in-depth discussion of implications and limitations. The comparison of $R_{0.1}$ with R_{1md} does not depend much on the sinking velocity (approximately the same value of c_s for both sinking velocities minimizes the W_d between these configurations, and matches their average travel distance and the surface area of the particle distributions optimally). We think that the exploration of more sinking velocities and saving times is out of scope for this manuscript and not necessary to answer the research question posed.

Reviewer 3

We would like to thank the reviewer for his/her careful reading and his/her constructive comments.

Please find our point by point reply and the changes in the manuscript below.

On behalf of the authors,

Peter Nooteboom

This manuscript considers the problem of identifying the source location of sinking particles found at the bottom of the ocean and examines the effect of coarse spatial (and to some extent temporal) resolution. The authors find that ocean general circulation models (OGCMs) at a resolution typical of state-of-the-art paleoclimate applications (1 degree) do not capture the distributions of potential source locations well. This can be remedied to some degree by using a Smagorinski parameterisation for the eddy effects that are not directly modeled.
I find the experiment reasonably designed and the manuscript overall well written, although the organization is a bit odd. Some of the discussion could also benefit from a broader perspective, considering alternative explanations, and some of the conclusions are stated too strongly. Therefore, I recommend this submission for publication subject to minor revisions, which should address the points below.
Detailed comments: 1. Experiment design: The case with highest spatial and temporal resolution was chosen as the reference case. This is reasonable, since one might anticipate its results to be closest to being accurate. However, it makes it difficult to disentangle effects of spatial and temporal resolutions. You might want to consider using R0.1m as the reference case, which can then be compared to (a) a case with the same spatial and different temporal resolution and (b) cases with different spatial and the same temporal resolution.

Author's response

The case with the highest spatial and temporal resolution was indeed chosen as a reference case because it is assumed to be the closest to reality. While we understand the point of the reviewer, we still like to make the comparison here with $R_{0.1}$ as reference case. Since anyone who wants to apply Lagrangian particles may often choose between 0.1° OGCMs (often written with output of daily order and 1° OGCMs (with monthly output), we regard the difference between these two configurations as most important.
The differences due to the temporal averaging are still shown by the differences between configurations $R_{0.1m}$ and $R_{0.1}$. The differences due to the resolution of the Eulerian model can still be inferred from configurations $R_{0.1m}$ and R_{1m}.

2. Abstract, 4th sentence: State-of-the-art OGCMS run on regional grids nowadays often provide output at much greater resolution, e.g. 1 km in space and 3-hourly in time is fairly common. The authors may want to specify here (in the abstract) that they are considering global models only. It would also be good to discuss implications of the present

findings for using a 10-km model with output every 5 days versus one with even higher spatial and temporal resolution. See also comment #22 below.

Author's response

Good point. We changed the manuscript accordingly.

Changes in manuscript

The sentence in the abstract is changed:
'In this study, we simulate the transport of sinking Lagrangian particles using low- and high-resolution global OGCMs, and assess the lateral transport differences resulting from the difference in spatial and temporal model resolution.'

We also added two paragraphs in the discussion to discuss the possibility of increasing the resolution more (see reviewer 2):
'Future work could investigate the sinking Lagrangian particles in other configurations with different OGCM resolutions. The model output of configuration $R_{0.1}$ could be coarsened to a 1° grid before the back-tracking analysis, to separate out the effects on the Lagrangian analysis of (a) a coarsened grid (see also [10, 15]) and (b) a lower resolution of the underlying Eulerian model. Moreover, particle trajectories could be sensitive to the vertical resolution of the OGCM (e.g. [4, 23]).
Some applications require different horizontal model resolutions than the configurations used in this paper. An OGCM of ~1km resolution has a better representation of the spatial/temporal submesoscale that can be important for sinking Lagrangian particles which represent the carbon flux to the ocean bottom [3]. Although the mesoscale flow contains most of the energy that is responsible for the tracer dispersion [24], submesoscale (1-20km) dynamics have proven to be of importance for the vertical advection of iron in specific regions with strong flow-bathymetric interactions [18].'

3. Abstract and Table 1: The phrase 5-daily is not entirely clear, as it could mean 5 times per day or once every 5 days. I recommend rephrasing it to clarify in these places. Further mentions should then be self-explanatory.

Author's response

Good point.

Changes in manuscript

We changed '5-daily' to 'once every 5 days' in the abstract and the table.

4. Lines 22 27: There are some other published studies that seem to be relevant in this context, including e.g. Wekerle et al., Frontiers Mar. Sci., 2018 (doi: 10.3389/fmars.2018.00407), which investigated sediment trap catchment areas using backward trajectories; Huntley et al., Ocean Model., 2011 (doi: 10.1016/j.ocemod.2010.11.001), which looked at the impact of both temporal and spatial resolution on the Lagrangian predictive skill of an

OGCM for neutrally buoyant particles; Ds et al., Ocean Model., 2011 (doi: 10.1016/j.ocemod.2011.05.005) which considered model-grid resolution impacts on dispersion rates; and Simonsen et al., J. Enc. Radioactivity, 2017 (doi: 10.1016/j.jenvrad.2017.06.002), which studied the effect of model resolution on the estimates of radionuclide transport.

Author's response

Thank you for these suggestions of similar studies that have been done.

Changes in manuscript

We added [29, 21, 5] to the other relevant literature on the topic in the introduction:
'The spatial resolution of the OGCM determines if the flow is eddying, which played an important role in simulations of sinking particles near the northern Gulf of Mexico [12] and in the Benguela region [13], and for passive tracers near Sellafield [21] and globally [5] ($0.25°$ *versus* $1°$ *resolution).'*
and
'The Lagrangian techniques are used to model the sinking particle trajectories in the modern ocean [30, 19, 20, 13, 28, 29], specifically for sinking microplankton [26, 14] and microplastic [7].'

Paper [10] is cited in the discussion, as they did an experiment with coarsening of the model output before the calculation of Lagrangian trajectories, which relates well to the points raised in the discussion:
'The model output of configuration $R_{0.1}$ *could be coarsened on* $1°$ *grid before the back-tracking analysis, to separate out the effects on the Lagrangian analysis in (a) a coarsened grid [10] and (b) a lower resolution of the underlying Eulerian model.'*

5. Line 59: Particles are released every three days over what time period? (The caption of Fig. 1 suggests 6 years.)

Author's response

It is indeed about 6 years, which is the time it takes until all released particles to reach the surface.

Changes in manuscript

The sentence is changed into:
'This means that we release particles at the bottom of the ocean every three days for more than a year, and compute their trajectories in the changing flow field back in time (similar to [14, 25, 29, 12]) until the particles reached the surface.'

6. Lines 106 109: This description is a bit unclear. First, there seem to be 9 different model configurations, depending on whether the choice for cs is considered part of the configuration. I suggest to use 4 main configurations, whereby R1m should consistently be chosen with the same value of cs (Given the results, I would suggest cs = 2), with

5 additional experiments to investigate the sensitivity of the results to cs. It is not helpful that in the figures R1m does not consistently refer to the same model configuration. Secondly, line 108 back-tracking from a single release location appears to contradict line 61, which specifies a grid of release locations. Reading further, it becomes clearer what is meant, but I recommend editing the description here. Also, is the 130 particles used at each location a typo? For releases every 3 days for 6 years (cf. caption of Fig. 1), there should be 730 releases.

Author's response

We already use four main configurations, as is shown in table 1 and explained in Line 106. In the text it is convenient to refer to a configuration with and one without diffusion added to the dynamics (the 'd' in the subscript stands for 'diffusion'), so we choose to stay with these four configurations. In the revised manuscript, we will consistently use a value of $c_s = 2$ in the plots.
The explanation on the back-tracking can indeed be more clear. We released a particle every three days, but not more than 130 particles per release location. At 6 m/day sinking velocity, the travel time can reach up to 4 years (see for example figure 7 of [14]), which implies that one has to wait until the sixth year before all particles reached the surface.

Changes in manuscript

No changes with respect to the decision of the configurations are made.
See comment 5 for changes with respect to the explanation of the analysis.

7. Lines 110 117, Fig. 1, and Results organization: On these lines and in the figure, the three metrics are listed in an order of increasing complexity. The lateral distance is a metric of an individual trajectory; the area metric describes the variability among launches at a single location; and the Wasserstein distance also takes into consideration where in space the distribution sits. This makes sense. But the Results are organized in the opposite order, starting with the most complex metric. I highly recommend changing that and following the order presented on lines 110 117, making it easier for the reader to interpret the additional elements captured in each metric.

Author's response

We understand why the Wasserstein distance is considered by the reviewer as the most complex metric.

Changes in manuscript

We changed the order of the results accordingly, consistent with the order of the metrics in Fig 1.

8. Line 127: Could you provide the range of typical travel times for each sinking velocity? In other words, what travel time is too short or long enough to see the eddy effects?

Author's response

The travel time also depends greatly on the depth of the release location. If the depth is 6km, the travel time could be respectively 2.8 and 0.7 years for sinking velocities 6m/day and 25m/day. However, the vertical ocean flow induce great variability in the travel time from one release location (see again figure 7 of [14]).

Changes in manuscript

We changed a sentence:
'The travel time of the particles is perhaps too short (at most a few years) for the errors in $R_{0.1m}$ to grow substantially, and remains ~~lower~~ smaller compared to R_{1m}.'

9. Line 130: Well, they are not the same; one can clearly distinguish them in Fig. 1d. It might be better to state that they are not significantly different. Also, if you consider these two curves to be the same, then so are the curves for the larger sinking velocity, which is not how they are discussed in the next paragraph.

Author's response

This was a typo. The lines are indeed not the same.

Changes in manuscript

We changed the sentence:
The global average W_d between $R_{0.1m}$ and the reference case ($W_d(R_{0.1}, R_{0.1m})$) is ~~the same as~~ slightly larger compared to the check of $R_{0.1}$ with itself (the global average $W_d(R_{0.1}, R_{0.1})$; dashed versus dotted in Fig 2d).'

10. Lines 131 134: I recommend explaining this earlier, maybe at the beginning of the Wasserstein distance comparison section, before the reader looks at the plot and starts wondering why that quantity isnt identically 0.

Author's response

We think that the explanation is clear as it is now. If a reader wonders why this value is unequal to 0 in the figure, the explanation is also included in the figure caption.

11. Line 136: There seems to be a strong difference between cs = 0 and cs positive. It is not clear that cs = 2 is significantly different from the other positive cs values. With that in mind, the statement that there is a minimizing value is misleading.

Author's response

We changed the sentence and hope that it is not experienced as misleading anymore.

Changes in manuscript

This sentence is changed:
'We find that ~~a value of c_s minimizes~~ any value of $c_s > 0$ reduces the W_d ~~(c_s = 2 for~~ by a similar amount, but the W_d is the smallest for $c_s = 2$ with both sinking speeds ~~w_f = 6, 25~~ $w_f = 6, 25$ m day^{-1} ~~; (~~(Fig 2d).'

12. Line 147: Consider showing a plot of the geographic distribution of the eddy kinetic energy in the high resolution model to support this statement.

Author's response

We added a figure in the supporting material with the mean surface eddy kinetic energy.

Changes in manuscript

The caption of the added supporting figure:
'Geographic plot of the time mean eddy kinetic energy at the surface. The eddy kinetic energy is defined as $\frac{1}{2}\overline{u' \cdot u'}$, where the bar denotes the time mean and u' the deviation from the time mean velocity vector u (so $u(\vec{x}, t) = \overline{u}(\vec{x}) + u'(\vec{x}, t)$).'

13. Lines 152 153: This sentence is unclear. More likely than what? Than in the other model? Than when the particles arent near each other? Since the latter is obvious, I assume you meant the former. A clearer wording might be Nearby particles are more likely to follow similar pathways in R0.1m than in R0.1. However, it is also not clear how this observation is relevant to the experiment at hand. The particles within each analysed cluster are not located close to each other, since they are separated in time. The link needs to be made more explicitly.

Author's response

Indeed, the particles are more likely to stay close to each other in $R_{0.1m}$ compared to $R_{0.1}$.

Changes in manuscript

We changed the wording of the paragraph:
'~~In terms of the~~ The surface area spanned by the particle distributions (Fig 1b) ~~,~~ is often smaller in $R_{0.1m}$ ~~does show a difference with~~ compared to $R_{0.1}$, as can be seen from the global ~~averages~~ average of this measure (Fig 3d). ~~Particles are more likely to follow~~ The lower surface area could be explained by the tendency of nearby particles to follow more similar pathways in $R_{0.1m}$ ~~if they are located close to each other~~ than in $R_{0.1}$ (see animation S2 in the supporting material) [16]. As a result, the particles will end up in clusters closer to each other at the surface. Hence, the surface area of the particle distributions is on average smaller in $R_{0.1m}$ compared to $R_{0.1}$.'

14. Line 155: At this point, the reader (or at least I) starts to wonder why R0.1m isnt included in Fig. 3. I suggest referencing Fig. 5 at this point.

Changes in manuscript

We added the reference to Fig 5 in the caption of Fig 3 and 4.

15. Fig. 3: Why is cs = 5 used here for panel (c)? It would help to pick a consistent value for all figures showing R1md. Also, Fig. 3d suggests that a value of 3.5 would be best to approximate R0.1, or a value of 2 to approximate R0.1m. This is consistent for both sinking velocities. Could you comment on this?

Author's response

The standard deviation of the noise is approximately between $6\sqrt{c_s}$ and $60\sqrt{c_s}$ (see lines 99-105 of the original manuscript). It implies that at exactly these values of c_s, the strength of the noise compares best to the scales of the eddies in $R_{0.1}$ and $R_{0.1m}$.

Changes in manuscript

We consistently used $c_s = 2$ in the revised manuscript figures.
We added a sentence to the manuscript:
'Interestingly, the value of c_s that approximates configuration $R_{0.1}$ ($c_s \approx 3.5$) and $R_{0.1m}$ ($c_s \approx 2$) best, is the same for both sinking velocities 6 and 25 m day^{-1}. These values of c_s must result in a similar scale of the fluctuations (σ_x, σ_y) in configurations $R_{0.1}$ and $R_{0.1m}$. It also indicates that, given c_s, the subgrid-scale parameterisation performance is similar for both sinking velocities. Nevertheless, the particle distributions match better with the reference configuration if the sinking velocity is higher (according to the W_d in Fig 2), because a lower particle travel time leads to less spread of the particle trajectories and a lower lateral displacement in all configurations.'

16. Lines 177 179: Maybe I misunderstand what is meant by a more smoothed pattern, but I dont think that smoothness explains this. Rather it is the lowering of the peaks in the Southern Ocean.

Author's response

The lowering of the peaks in the Southern Ocean is what we mean with 'less extreme.'

Changes in manuscript

We changed these sentences:
'The average lateral displacement becomes globally ~~more smoothed and~~ less extreme (especially the Southern Ocean peaks are lower) if the Smagorinsky diffusion is added to the flow dynamics in R_{1md} compared to R_{1m} (Fig 4c). The ~~more smoothed~~ less extreme pattern of the travel distances explains why the globally averaged lateral travel distance is minimal at $c_s = 0.25$ (for $w_f = 6$ m day^{1} in Fig 4d), and not at $c_s = 0$.'

17. Lines 189 190: See comment #13 it is not clear that this is relevant. The explanation might be instead that particles dont travel as far and hence dont spread out as

much. That would be supported by Fig. 5b. This, in turn, is a result of the velocities generally having lower peaks due to longer temporal averaging.

Author's response

These lines refer to the non-eddying configuration, not to the eddying configuration with monthly averaged model output. Fig 5 does not include any information on the non-eddying configurations, only on the difference between the reference configuration with the eddying configuration with monthly model output.

Changes in manuscript

To make clear that these lines are about the differences between the eddying reference configuration $R_{0.1}$ and the non-eddying configuration, we add 'non-eddying' before R_{1m}.

18. Lines 193 194: The two panels of Fig. S1 should opposite behavior in a very similar location. In one case, the blue particles are closer together than the red ones, in the other they are farther apart. This needs to be addressed in the text.

Changes in manuscript

It is now addressed in the text:
'This could result in notably different back-tracked particle distributions, especially if the shear of the flow field is high (see for instance the location $45.5°S$*,* $39.5°E$ *on plankton-drift.science.uu.nl or Fig S1 for two similar locations with opposite behaviour).'*

19. Lines 197 200: This is not entirely true. Fig. 4 shows that in some regions (mainly the Southern Ocean), individual trajectories disperse more in the absence of eddies.

Author's response

With 'disperse less' we meant here that the trajectories disperse less in comparison with each other. In Fig 4, one can see that the particles travel further from their release location in some areas (such as the Southern Ocean), yet they originate from surface locations which are close to each other (i.e. the particle distribution consists of particles which are located close to each other; as can be seen in Fig 3b).

Changes in manuscript

We changed
'disperse less'
into
'spread less.'

20. Fig. 6: It seems that R1m appears mostly in black rather than yellow, because only the outlines of the markers are visible.

Author's response

Agreed.

Changes in manuscript

The dots are made entirely black.

21. Lines 242 247: These conclusions are overstated. I am not convinced that the mean flow field in coarser models is wrong in most areas (line 242). At a minimum, this claim needs a reference. In addition, in Fig. 3 and Fig. 4, it looks like the diffusion is sufficient in most areas! So, I dont understand the justification for the claim in lines 246 247. Lastly, these impacts seem to be highly dependent on the sinking velocity, which needs to be acknowledged.

Author's response

The lack of low resolution models to represent the time-mean flow was discussed in the introduction of the manuscript, and supported with several references (e.g. [9, 2, 8]).
Fig 3 and 4 show that the addition of diffusion in the low resolution model can increase the surface area of the particle distributions, up to a point where they match well with the reference high-resolution configuration. However, the Wasserstein distance of Fig 2 shows that the particle distributions are still distant from the reference configuration. This result highlights that the coarse models do show a different surface origin location compared to the high resolution models.
The differences of the particle distributions between the configurations are indeed influenced by the sinking velocities. However, this does not imply that the effects of eddies is easier to capture at a higher sinking velocity. In contrast, a higher sinking speed leads to a lower particle travel time in all configurations. As a consequence, the particle distributions will be less spread out and the lateral travel distance will be lower, which is easier to represent.

Changes in manuscript

We rephrased this paragraph in the discussion:
'Overall, the sinking Lagrangian particles give unrealistic results in the non-eddying models, because (1) the back-tracked distributions show too little spread due to the absence of ocean eddies and (2) these models often do not capture the mean flow fields correctly ~~at most locations.~~ (as shown in e.g. [9, 2, 8]). Lateral stochastic diffusion in the low-resolution configuration (re-)introduced part of the eddy fluctuations and hence increased the relative dispersion of particle trajectories~~and the spread of the backtracked~~ , and increased the lateral travel distance (Fig 2) and the spread (Fig 3) of the back-tracked particle distributions. Hence this method is promising for locations where the ~~OGCMs are able to~~ low resolution OGCMs capture the mean flow field ~~.~~ well. However, the particle distributions are often distant from the distributions of the reference configuration, as is shown from the Wasserstein distance in Fig 5, which implies that the surface origin location is different between the coarse and high resolution models. The Smagorinsky diffusion was insufficient to parameterise the eddies in most areas.'

We also added a sentence in the results section (Lines 156-162 of old manuscript), to make clear that, given a value of c_s, the performance of the eddy parameterisation is similar for both sinking velocities. However, the particle distributions match better at a higher sinking velocity w_f due to the lower travel times. See comment 15 for the paragraph which includes this sentence.

22. Lines 248 253: Will eddying (i.e., a resolution of 10 km) suffice? Why wouldnt we expect this effect to continue as the model resolution is increased and eddies of smaller and smaller size are being resolved?

Author's response

Indeed, a comparison of the back-tracking analysis with Eulerian models of higher horizontal resolution is interesting. Especially, it can be interesting for the modelling of sinking particles in the present-day.
The two model resolutions (1° and 0.1° horizontally) in this manuscript are the highest resolutions that are typically used globally in palaeo models and present-day, respectively. Due to long spin-up times, palaeo simulations at 10km resolution do not exist yet, and palaeo simulations at 2km resolution require even more computational time.

Changes in manuscript

The paragraph which is added to the discussion addresses this point (see comment 2 for the specific text).

23. Lines 256 257: What does it mean that these models do not produce a flow field that is representative? I would imagine that paleo modelers would consider their models to represent the time period being modeled.

Author's response

These sentences should indeed be rephrased. These models are to some extend representative to the time period, but they do not have the eddying characteristics and do not solve the mean flow correctly as is discussed.

Changes in manuscript

We changed:
'OGCMs used in most paleo studies ~~will~~ lack the eddying flow characteristics and do not generate a ~~flow field which is locally representative~~ locally representative time mean flow for the time period of interest. Since Lagrangian particles use the local flow field, ~~the Lagrangian technique requires~~ they require eddying paleoceanographic models that ~~are representative~~ better represent the time mean flow for the considered time period.'

Typos and such:

- **Abstract, 4th sentence: It should be on the order of...**

- **Line 35: There should be no comma before the and.**
- **Line 61: on a 1o x 1o global grid**
- **Line 63: Since this isnt being shown in the present study, past tense would be better: has been shown to be a proper way.**
- **Line 68: Again, it should be on the order of...**
- **Fig. 1 caption, last line: The of following the parentheses is superfluous.**
- **Fig. 2 caption, last sentence: ... is shown between the particle distributions of the same...**
- **Fig. 2(c) title: diffusion is misspelled.**
- **Line 150: The reference should be to Fig. 1c (not b).**
- **Captions for Fig. 3 and Fig. 4: The subscript in cs isnt written as a subscript. R1m is missing the m. For Fig. 4 caption, it should be Globally averaged.**
- **Line 162: It should be monotonically, not monotonously.**
- **Lines 175 and 177: Replace more smoothed with smoother.**
- **Line 187: Replace more clear with clearer.**
- **All the global geographic plots in Fig. 2 4 have incorrect longitude labels. Also, consider using the same view for all global geographic plots, either that used in Fig. 5 or that used for the other figures.**
- **Fig. 6 caption, first line: R1m is missing the m. Second to last line: Are the depths really identical for panel (c), or is this a typo? Period missing at the end of the caption.**
- **References:**
  - **Please include DOIs wherever possible.**
  - **Ref. 4 should have second author 'Nothing E-M'.**
  - **Ref. 5 is missing the authors initial.**
  - **Ref. 8 should have last author 'van Sebille, E'; similar for Ref. 16.**
  - **Ref. 13 should capitalize Garca.**
  - **Ref. 14 accidentally repeats part of the title.**
  - **Ref. 44: If this is a book title, it shouldnt be abbreviated.**
  - **Ref. 50 should capitalize Kemper.**

Author's response

Thank you for noticing these typos. For the Fig. 6 caption: The depth is indeed the same for panel (c).

Changes in manuscript

The typos have been checked and are changed accordingly.

References

[1] Iván J Alonso-González, Javier Arístegui, Cindy Lee, Anna Sanchez-Vidal, Antoni Calafat, Joan Fabrés, Pablo Sangrá, Pere Masqué, Alonso Hernández-Guerra, and Veronica Benitez-Barrios. Role of slowly settling particles in the ocean carbon cycle. *Geophys. Res. Lett.*, 37:1–5, 2010.

[2] P. Berloff, A.McC. Hogg, and W. Dewar. The Turbulent Oscillator : A Mechanism of Low-Frequency Variability of the Wind-Driven Ocean Gyres. *Am. Meteorol. Soc.*, 37:2363–2386, 2007.

[3] Philip W. Boyd, Claustre Herve, Marina Levy, David A Siegel, and Thomas Weber. Multi-faceted particle pumps drive carbon sequestration in the ocean. *Nature*, 568:327–335, 2019.

[4] Annalisa Bracco, Jun Choi, Jaison Kurian, and Ping Chang. Vertical and horizontal resolution dependency in the model representation of tracer dispersion along the continental slope in the northern Gulf of. *Ocean Model.*, 122(December 2017):13–25, 2018.

[5] Kristofer Döös, Volfango Rupolo, and Laurent Brodeau. Dispersion of surface drifters and model-simulated trajectories. *Ocean Model.*, 39:301–310, 2011.

[6] Peter R Gent. The Gent McWilliams parameterization : 20 / 20 hindsight. *Ocean Model.*, 39:2–9, 2011.

[7] Britta D Hardesty, Joseph Harari, Atsuhiko Isobe, Laurent Lebreton, Nikolai Maximenko, Jim Potemra, Erik Van Sebille, A Dick Vethaak, and Chris Wilcox. Using Numerical Model Simulations to Improve the Understanding of Micro-plastic Distribution and Pathways in the Marine Environment. *Front. Mar. Sci.*, 4(March):1–9, 2017.

[8] Matthew W Hecht and Richard D. Smith. A Review of North Atlantic Ocean Modeling in an Eddying Regime. *Geophys. Monogr. Ser.*, 177:108–109, 2013.

[9] William R. Holland. The role of mesoscale eddies in the general circulation of the ocean - Numerical experiments using a wind-driven quasi-geostrophic model. *Am. Meteorol. Soc.*, 8:363–392, 1978.

[10] Helga S Huntley, B.L. Lipphardt Jr., and A.D. Kirwan jr. Lagrangian predictability assessed in the East China Sea. *Ocean Model.*, 36(1-2):163–178, 2011.

[11] Merel Kooi, Egbert H Van Nes, Marten Scheffer, and Albert A Koelmans. Ups and Downs in the Ocean: Effects of Biofouling on Vertical Transport of Microplastics. *Environ. Sci. Technol.*, 51:7963–7971, 2017.

[12] Guangpeng Liu, Annalisa Bracco, and Uta Passow. The influence of mesoscale and submesoscale circulation on sinking particles in the northern Gulf of Mexico. *Elem. Sci. Athropocene*, 6(36), 2018.

[13] Pedro Monroy, Gabor Drótos, Emilio Hernández-garcía, and Cristóbal López. Spatial Inhomogeneities in the Sedimentation of Biogenic Particles in Ocean Flows: Analysis in the Benguela Region. *J. Geophys. Res. Ocean.*, 124(May):1–19, 2019.

[14] Peter D Nooteboom, Peter K Bijl, Erik van Sebille, Anna S. Von Der Heydt, and Henk A. Dijkstra. Transport Bias by Ocean Currents in Sedimentary Microplankton Assemblages : Implications for Paleoceanographic Reconstructions. *Paleoceanogr. Paleoclimatology*, 34, 2019.

[15] Nathan Putman and Ruoying He. Tracking the long-distance dispersal of marine organisms : Sensitivity to ocean model resolution. *J. R. Soc. Interface*, 10(February 2016), 2013.

[16] Xuerong Qin, Erik van Sebille, and Alexander Sen Gupta. Quantification of errors induced by temporal resolution on Lagrangian particles in an eddy-resolving model. *Ocean Model.*, 76:20–30, 2014.

[17] J S Riley, R Sanders, C Marsay, F A C Le Moigne, E P Achterberg, and A J Poulton. The relative contribution of fast and slow sinking particles to ocean carbon export. *Global Biogeochem. Cycles*, 26:1–10, 2012.

[18] Isabella Rosso, Andrew Mcc, Richard Matear, and Peter G Strutton. Deep-Sea Research I Quantifying the in fl uence of sub-mesoscale dynamics on the supply of iron to Southern Ocean phytoplankton blooms. *Deep. Res. Part I*, 115:199–209, 2016.

[19] D A Siegel and W.G. Deuser. Trajectories of sinking particles in the Sargasso Sea: modeling of statistical funnels above deep-ocean sediment traps. *Deep. Res. 1*, 44(9):1519–1541, 1997.

[20] D A Siegel, E Fields, and K O Buesseler. A bottom-up view of the biological pump : Modeling source funnels above ocean sediment traps. *Deep. Res. Part 1*, 55:108–127, 2008.

[21] Magne Simonsen, Øyvind Saetra, Pål Erik, Ole Christian, Hilde Kristin, Brit Salbu, Hilde Elise Heldal, and Justin P Gwynn. The impact of tidal and mesoscale eddy advection on the long term dispersion of 99Tc from Sellafield. *J. Environ. Radioact.*, 177:100–112, 2017.

[22] J. Smagorinsky. General circulation experiments with the primitive equations. *Mon. Weather Rev.*, 91(3):99–163, 1963.

[23] K D Stewart, A Mcc Hogg, S M Griffies, A P Heerdegen, M L Ward, P Spence, and M H England. Vertical resolution of baroclinic modes in global ocean models. *Ocean Model.*, 113:50–65, 2017.

[24] Takaya Uchida, Dhruv Balwada, Ryan Abernathey, Galen McKlinley, Shafer Smith, and Marina Levy. The Contribution of Submesoscale over Mesoscale Eddy Iron Transport in the Open Southern Ocean. *J. Adv. Model. earth Syst.*, 11:3934–3958, 2019.

[25] Erik van Sebille, Stephen M. Griffies, Ryan Abernathey, Thomas P. Adams, Pavel Berloff, Arne Biastoch, Bruno Blanke, Eric P. Chassignet, Yu Cheng, Colin J. Cotter, Eric Deleersnijder, Kristofer Döös, Henri F. Drake, Sybren Drijfhout, Stefan F. Gary, Arnold W. Heemink, Joakim Kjellsson, Inga Monika Koszalka, Michael Lange, Camille Lique, Graeme A. MacGilchrist, Robert Marsh, C. Gabriela Mayorga Adame, Ronan

McAdam, Francesco Nencioli, Claire B. Paris, Matthew D. Piggott, Jeff A. Polton, Siren Rühs, Syed H.A.M. Shah, Matthew D. Thomas, Jinbo Wang, Phillip J. Wolfram, Laure Zanna, and Jan D. Zika. Lagrangian ocean analysis: Fundamentals and practices. *Ocean Model.*, 121(July 2016):49–75, 2018.

[26] Erik van Sebille, Paolo Scussolini, Jonathan V. Durgadoo, Frank J. C. Peeters, Arne Biastoch, Wilbert Weijer, Chris Turney, Claire B. Paris, and Rainer Zahn. Ocean currents generate large footprints in marine palaeoclimate proxies. *Nat. Commun.*, 6:6521, 2015.

[27] M. Villa-Alfageme, F.C. Soto, E. Ceballos, S.L.C. Giering, F.A.C Le Moigne, S. Henson, J.L. Mas, and R.J. Sanders. Geographical, seasonal, and depth variation in sinking particle speeds in the North Atlantic. *Geophys. Res. Lett.*, 43:8609–8616, 2016.

[28] Joanna Waniek, Wolfgang Koeve, and Ralf D Prien. Trajectories of sinking particles and the catchment areas above sediment traps in the northeast Atlantic Trajectories of sinking particles and the catchment areas. *J. Mar. Res.*, 58:983–1006, 2000.

[29] Claudia Wekerle, Thomas Krumpen, Tilman Dinter, Wilken-jon von Appen, Morten Hvitfeldt Iversen, and Ian Salter. Properties of Sediment Trap Catchment Areas in Fram Strait : Results From Lagrangian Modeling and Remote Sensing. *Front. Mar. Sci.*, 5(November), 2018.

[30] Qiu Zhongfeng, A M Doglioli, and F Carlotti. Using a Lagrangian model to estimate source regions of particles in sediment traps. *Sci. China Earth Sci.*, 57(10):2447–2456, 2014.
