# Supplementary material for: Resolution dependency of sinking Lagrangian particles in ocean general circulation models
Source: PLoS One. 2020 Sep 10;15(9):e0238650. doi: 10.1371/journal.pone.0238650 (PMC7482921; doi:10.1371/journal.pone.0238650)
Supplement: Attachment — Submitted filename: reviewer_response.pdf [file pone.0238650.s006.pdf]

Reviewer 1

We would like to thank the reviewer for his/her careful reading and his/her constructive comments.

Please find our point by point reply and the changes in the manuscript below.

On behalf of the authors,

Peter Nooteboom

Reviewer comment

This revised ms. is much improved in making clear what is the goal, and how it has been addressed. The paper is now clearly addressed to what are expected to be naive paleoceanographers who take model results much too seriously. As such, I would expect the authors to have sent this paper to some place like Quaternary Science Reviews, but that is their business (and that of the Editor of PLOS ONE). Assuming everyone agrees that the journal is appropriate, the paper can be accepted in principle, but there exist a number of amplifications that would be very useful if the readership really is thought to be uninformed about numerical ocean models.

Author's response

The manuscript is intended for a broad audience, such as modellers, sedimentologists, environmental marine scientists, biologists, paleontologists and oceanographers, which is why it does fit into PLOSone.

Reviewer comment

Readers need to be told that physical oceanographers distinguish between "eddy-permitting" models and "eddy-resolving" ones. This distinction would be crucial to anyone taking the paper seriously, but it is not even mentioned. Are internal tides, internal waves, submesoscales, of no concern?

Author's response

All of the raised points are mentioned in the discussion now.

Changes in manuscript

'~~*Some applications require different horizontal model resolutions than the configurations used in this paper.*~~ *When models do not resolve the so-called internal Rossby deformation radius (about 50 km at midlatitudes), no eddies can be represented. When the model grid scale is only slightly smaller than the deformation radius, say 25 km at midlatitudes, eddies form but their interaction is not fully captured; such a model is called 'eddy permitting.' Only for models at about 1 km horizontal midlatitude resolution (so-called eddy-resolving models), eddy interactions are fully resolved. The 10 km resolution POP model, as is used here ($R_{0.1}$), is*

therefore often called 'strongly eddying.' An OGCM of ∼1km resolution also has a better representation of the spatial/temporal submesoscale that can be important for sinking Lagrangian particles which represent the carbon flux to the ocean bottom [2]. Although the mesoscale flow contains most of the energy that is responsible for the tracer dispersion [11], submesoscale (1-20km) dynamics have proven to be of importance for the vertical advection of iron in specific regions with strong flow-bathymetric interactions [10]. Future work could analyse the transport of sinking particles in models with higher resolutions, and with models which better solve internal tides [13] or improved interaction of the bottom-flow with topography [7].

Reviewer comment

The Wasserstein distance is fundamental to their results, but the paper has no explanation except for a reference number. Explain.

Author's response

The Wasserstein distance is explained in the method section:
'The Wasserstein distance is the minimum distance that one has to displace the particles resulting from one simulation (along the dashed lines in Fig 1d) to transform it into another particle distribution (and is calculated with [1]).'

Reviewer comment

Even in simple laminar flows, particle trajectories are known to be chaotic (e.g., Regier and Stommel, 1979 PNAS). Why is that not an issue here?

Author's response

Because of the chaotic nature of particle trajectories, we do not analyse single trajectories, but rather an ensemble of trajectories and its statistics, which is often done in Lagrangian analysis [12].

Changes in manuscript

We changed the method section:
'This means that we release particles at the bottom of the ocean every three days for more than a year, and compute their trajectories in the changing flow field back in time (similar to [8, 12, 14, 4]) until the particles reached the surface. We stop a particle if it reaches 10m depth. The particles are released on a $1^\circ \times 1^\circ$ global grid. The resulting particle distributions allow us to investigate the statistics of particle ensembles, rather than single trajectories. Particle ensemble statsistics are often used in Lagrangian analysis [12], because of the chaotic nature of the particle trajectories [9]. '

Reviewer comment

It is likely true that the vertical velocity in the ambient fluid is negligible compared to the sinking velocities. But in upwelling zones? (See e.g., Liang JGR 2018) Again, useful information for non-modelers.

Author's response

The sinking velocities of the ambient fluid are often of importance, as was shown in figure 7 of [8].

Changes in manuscript

We added a sentence in the method section, to make this clear:
'The vertical part of the flow $\vec{v}$ can be relevant compared to the particle sinking velocity w_f (see Fig 7 in [8]).'

Reviewer comment

The complexity now known about near-bottom flows in the presence of topography needs to be at least mentioned. A large recent literature on this subject and probably very important in the particles landing in sedimentary regions.

Author's response

Agreed.

Changes in manuscript

This topic is mentioned in the discussion now with a reference:
'Although the mesoscale flow contains most of the energy that is responsible for the tracer dispersion [11], submesoscale (1-20km) dynamics have proven to be of importance for the vertical advection of iron in specific regions with strong flow-bathymetric interactions [10]. Future work could investigate the transport of sinking particles in models with higher resolutions, and with models which better represent internal tides [13] or improved interaction of the bottom-flow with topography [7]. '

Reviewer comment

The authors keep referring to the "mean" flow. Is there a definable mean flow? Over what time-scale? Same everywhere?

Author's response

The mean flow here is the mean flow over the total simulation period (over several years).

Changes in manuscript

We added this the first time that 'mean flow' is mentioned:
'For example, for a location in the Antarctic Circumpolar Current (ACC, Fig 6b), the mean flow field (averaged over 6 years) in R_{1m} is similar to the mean flow field in $R_{0.1}$.'

Reviewer comment

The bolus velocity is mentioned on P. 14. If it was ever defined, I missed it.

Author's response

The bolus velocity was defined in the method section, together with references to papers about the GM parameterisation (see lines 95-100 of the revised manuscript).

Changes in manuscript

None.

Reviewer comment

Can something be said about the skill of the reference model in reproducing the modern circulation elements, such as the eddy intensities, spatial scales, etc.?

Author's response

Certainly.

Changes in manuscript

We added a sentence in the method section, with a reference to a study that compares several ocean models, among which POP, with the present-day observed ocean circulation:
'The eddying POP version has a reasonably good representation of the modern circulation compared to other models at the same resolution [6].'

Reviewer comment

Consider comparing Fig. 3 to the "ventilation" Fig. 2 of Gebbie and Huybers (GRL 2011). Just needs one sentence.

Author's response

The surface area of backtracked particle distributions is expected to be lower in downwelling areas compared to upwelling areas. As such, one might expect the surface area in Fig 3 to be lower (higher) in areas with a relative large (low) surface origin of water volume in Fig 2 of the Gebbie and Huybers paper. One can observe this in sinking regions such as northeast of Iceland and in the Ross Sea, and in upwelling regions such as the equator.

Changes in manuscript

'Locally, the surface area of the particle distributions shows a different pattern in R_{1md} compared to the reference $R_{0.1}$ (Fig 3a vs c). In contrast to the magnitude of the noise, the direction of the noise vector does not depend on the flow field (it is horizontally isotropic). Therefore, the surface area of the particle distributions in configuration R_{1md} is overestimated in the tropics compared to the reference configuration $R_{0.1}$, where the flow is mostly zonal. Interestingly, this measure remains low in areas with sinking waters for both configurations $R_{0.1}$ and $R_{0.1md}$, such as the Ross Sea and the Weddell sea (see Fig 2 in [3]). .'

Reviewer comment

Is ”rectification” (P. 7) the right word? Comes from radio engineering and implies self-interaction producing a mean or low frequency.

Author’s response

‘Rectification’ is often used in the literature in this context.

Changes in manuscript

We added a reference to a study about mesoscale eddy parameterisation that uses the word: *‘However, it is well known that non-eddying OGCMs do not get the mean flow field right in all of the locations, because the eddies influence the mean flow field through rectification [5].’*

Reviewer comment

Put horizontal dimensions scales on Fig. 1.

Author’s response

The horizontal dimensions were left out on purpose in this illustration. The addition of horizontal dimension means that the figure should include gridlines, which distracts the reader from the purpose of the plot, which is to illustrate the three measures that are used in the manuscript.

Reviewer 3

The authors have done a good job addressing my previous concerns. I now recommend the manuscript for publication.

We would like to thank the reviewer again for his/her careful reading and his/her constructive comments.

On behalf of the authors,

Peter Nooteboom

References

[1] Flamary R, Courty N. POT Python Optimal Transport library; 2017. Available from: https://github.com/rflamary/POT.

[2] Philip W. Boyd, Claustre Herve, Marina Levy, David A Siegel, and Thomas Weber. Multi-faceted particle pumps drive carbon sequestration in the ocean. *Nature*, 568:327–335, 2019.

[3] Geoffrey Gebbie and Peter Huybers. How is the ocean filled ? *Geophys. Res. Lett.*, 38(February):1–5, 2011.

[4] Guangpeng Liu, Annalisa Bracco, and Uta Passow. The influence of mesoscale and submesoscale circulation on sinking particles in the northern Gulf of Mexico. *Elem. Sci. Athropocene*, 6(36), 2018.

[5] Piergianluca Porta Mana and Laure Zanna. Toward a stochastic parameterization of ocean mesoscale eddies. *Ocean Model.*, 79:1–20, 2014.

[6] J. McClean, S. Jayne, M. Maltrud, and D. Ivanova. The Fidelity of Ocean Models With Explicit Eddies. In M.W. Hecht and H. Hasumi, editors, *Ocean Model. an Eddying regime*, chapter 2, pages 149–164. American Geophysical Union, Washington, DC, 2008.

[7] Elda Miramontes, Pierre Garreau, Matthieu Caillaud, Gwenael Jouet, Romain Pellen, F Javier Hernández-molina, Michael A Clare, and Antonio Cattaneo. Contourite distribution and bottom currents in the NW Mediterranean Sea : Coupling seafloor geomorphology and hydrodynamic modelling. *Geomorphology*, 333:43–60, 2019.

[8] Peter D Nooteboom, Peter K Bijl, Erik van Sebille, Anna S. Von Der Heydt, and Henk A. Dijkstra. Transport Bias by Ocean Currents in Sedimentary Microplankton Assemblages : Implications for Paleoceanographic Reconstructions. *Paleoceanogr. Paleoclimatology*, 34, 2019.

[9] Lloyd Regier and Henry Stommel. Float trajectories in simple kinematic flows. *Proc. Natl. Acad. Sci.*, 76(10):4760–4764, 1979.

[10] Isabella Rosso, Andrew Mcc, Richard Matear, and Peter G Strutton. Deep-Sea Research I Quantifying the in fl uence of sub-mesoscale dynamics on the supply of iron to Southern Ocean phytoplankton blooms. *Deep. Res. Part I*, 115:199–209, 2016.

[11] Takaya Uchida, Dhruv Balwada, Ryan Abernathey, Galen McKlinley, Shafer Smith, and Marina Levy. The Contribution of Submesoscale over Mesoscale Eddy Iron Transport in the Open Southern Ocean. *J. Adv. Model. earth Syst.*, 11:3934–3958, 2019.

[12] Erik van Sebille, Stephen M. Griffies, Ryan Abernathey, Thomas P. Adams, Pavel Berloff, Arne Biastoch, Bruno Blanke, Eric P. Chassignet, Yu Cheng, Colin J. Cotter, Eric Deleersnijder, Kristofer Döös, Henri F. Drake, Sybren Drijfhout, Stefan F. Gary, Arnold W. Heemink, Joakim Kjellsson, Inga Monika Koszalka, Michael Lange, Camille Lique, Graeme A. MacGilchrist, Robert Marsh, C. Gabriela Mayorga Adame, Ronan McAdam, Francesco Nencioli, Claire B. Paris, Matthew D. Piggott, Jeff A. Polton, Siren

Rühs, Syed H.A.M. Shah, Matthew D. Thomas, Jinbo Wang, Phillip J. Wolfram, Laure Zanna, and Jan D. Zika. Lagrangian ocean analysis: Fundamentals and practices. *Ocean Model.*, 121(July 2016):49–75, 2018.

[13] Clément Vic, Alberto C Naveira Garabato, J A Mattias Green, Amy F Waterhouse, Zhongxiang Zhao, Angélique Melet, Casimir De Lavergne, Maarten C Buijsman, and Gordon R Stephenson. Deep-ocean mixing driven by small-scale internal tides. *Nat. Commun.*, 10(2099), 2019.

[14] Claudia Wekerle, Thomas Krumpen, Tilman Dinter, Wilken-jon von Appen, Morten Hvitfeldt Iversen, and Ian Salter. Properties of Sediment Trap Catchment Areas in Fram Strait : Results From Lagrangian Modeling and Remote Sensing. *Front. Mar. Sci.*, 5(November), 2018.
